# The Diagnosis and Treatment of Erosion of the Cervix Uteri

**Published:** 1910-03-26

**Authors:** C. Willett Cunnington


### The General Practitioner's Column.

[Contributions to this Column are invited, and, if accepted, will be paid for.]

### THE DIAGNOSIS AND TREATMENT OF EROSION OF THE CERVIX UTERI.

By C. WILLETT CUNNINGTON, M.B., B.C.Cantab.

Erosion of the cervix is one of those minor maladies of women which are more commonly discovered in hospital than in private practice.

No doubt this is because in the former more patients are examined per vaginam as a matter of routine than is either possible or convenient in the latter. Hence, while cervical erosions are regarded by the gynæcologist as "every day" affairs, to the general practitioner they are not so common that he has much experience of them; it may, therefore, be useful to have a brief description of this condition and of the methods by which it should be treated.

It is not necessary to dwell upon the pathology of erosions, beyond reminding the reader that they are not, strictly speaking, ulcerations, but flat adenomatous growths. It is wiser, however, in describing to a patient the nature of her disorder, to retain the popular term with which she is probably familiar, and to avoid the word "growth," indissolubly connected in her mind with "cancer." An erosion of the cervix may be associated with symptoms so marked that it is on their account that the patient consults a doctor; yet in many cases they are so trifling that the condition is only discovered by chance examination.

This difference is probably due to the fact that in some the cervix and cervical canal are inflamed as well, and the more noticeable symptoms are in reality produced by the inflammatory condition. When there is no inflammation there are seldom symptoms sufficient to attract the patient's attention.

In the former group of cases we find the patient complaining of pain in the back, and on more exact investigation this is discovered to mean a triangle covering the last lumbar vertebra, an area which is often tender as well. When questioned she will add that the pain is worse after long standing, that it extends downward into the thighs, and she may point to the ovarian regions as being painful as well. Associated with the pain, which may be quite severe or may be slight, there is a vaginal discharge, either white or yellow, and often tinged with blood. In married women there is sometimes dyspareunia. Such pain as there is will be more noticeable just before menstruation and is aggravated by constipation.

The discharge may be irritating, and often it is on that account that the patient asks for relief. One sees sometimes young unmarried women to whom the local irritation is a source of much distress and worry. It is a factor in the production of "bad habits," and should not be forgotten in the treatment of these difficult cases. The natural objection to the vaginal examination of virgins leads, in private practice, to the overlooking of a condition which can hardly be cured except by local applications. It is important to bear in mind that erosions are common enough in unmarried girls, and a certain proportion of these require local treatment. It needs a discreet judgment to decide when to "leave matters alone" and when to attack the cause direct. In private practice the error is too often on the side of unwise neglect.

On vaginal examination the well known "velvety" feel of the cervix is recognised unless the erosion is small and centred round the os, so that a view of the cervix through a Ferguson's speculum is desirable, in addition to digital examination.

In appearance the ordinary erosion has a dull beefy red look; its margin is sharply defined from the paler, smoother surface of the surrounding cervix. Even where the cervix is inflamed its mucous membrane, although vivid, remains shiny, and the distinction is conspicuous. An opaque white or yellowish discharge emanates from the external os, and may require mopping away before a clear view is obtained. To cleanse the canal and surface of the cervix nothing is so good as a wool mop soaked in a saturated solution of sodium bicarbonate.

The surface of the erosion, especially if it be of the papillomatous type, can be made to bleed slightly by the touch of a probe, but never so readily as can a carcinoma, nor will its substance be so easily disintegrated by the point of the probe. However the differential diagnosis is sometimes not easy by inspection and touch alone. If there be any doubt it is a simple matter to snip off a small portion for microscopical examination. To temporise with the view of "watching the effect of treatment" is a serious mistake; nor should one be too much biassed by the youth of the patient, as carcinoma of the cervix may occur even in the early twenties.

In parous women it is needful to distinguish between an erosion, which is flat, and a gaping torn cervix flattened out for the moment by the pressure of a speculum. Examination with the finger will, in the latter, reveal the torn edge of the cervix. We have also to determine in women who have had children whether the erosion is complicated by uterine subinvolution, and in all cases whether there be endometritis or displacement of the uterus.

An erosion may be aggravated by the presence of a pessary, and in these circumstances the instrument should not be used until the cervix is restored to a healthy condition. Suppose we have to deal with an uncomplicated erosion, we have to decide whether it needs any treatment at all, especially if the patient be a virgin.

In married women erosions may perhaps be a cause of sterility, and should therefore be treated even though symptoms be insignificant. A speculum is passed, the patient lying either on her side or on her back, and the cervix and its canal cleansed of mucus in the way described. A dressed Playfair's probe, dipped in iodised phenol (iodine gr. 40 in pure carbolic acid ʒj) is passed into the canal as far as, but not beyond, the internal os. By vigorous rotation the interior of the canal is denuded of its surface layer of cells. The caustic is then rubbed over the surface of the erosion, to remove the columnar epithelium of the adenomatous growth. The surface is whitened by the carbolic acid. Any excess of caustic is mopped up and a tampon of wool soaked in ichthyol (10 per cent.) in glycerine is passed up the speculum and the latter is withdrawn. A tape attached to the waist of the tampon hangs just outside the vaginal orifice. By means of this the patient is able to pull out the tampon after 24 hours.

She is instructed to douche herself with some astringent solution (such as zinc sulphate gr. xx. and alum gr. 40, aq. Oj), night and morning. The painting process should be repeated twice a week to begin with, and later less often.

The erosion should disappear in a month's time, the healthy pavement epithelium gradually encroaching on the diseased area, until at length the whole is found to be smooth and shiny. Excessive applications of caustic to the interior of the cervical canal may lead to constriction of its lumen by scar tissue; therefore this part of the treatment should be dropped as soon as the cervical discharge ceases. If the discharge comes from an unhealthy endometrium, curettage is needed before the treatment of the erosion is commenced. Similarly a marked vaginitis must be subdued by appropriate means before much effect can be expected of applications to an erosion.

In cases of gonorrhœal origin, the iodised phenol should be replaced by a 10 per cent. solution of protargol, and this can be well rubbed in over the whole vaginal mucous membrane as well as into the cervical canal.

Even in cases of uncomplicated erosion it is not wise to promise a cure, as a small proportion remain obstinate, no matter what treatment be adopted. These patients may be comforted by the assurance that the condition has nothing to do with cancer, nor are they especially prone to develop cancer, a fear of which is possibly ever present in their minds.

